# Supplementary material for: Attraction, Dynamics, and Phase Transitions in Fire Ant Tower-Building
Source: Front Robot AI. 2020 Mar 4;7:25. doi: 10.3389/frobt.2020.00025 (PMC7806095; doi:10.3389/frobt.2020.00025)
Supplement: Supplementary file 9 [file Data_Sheet_1.pdf]

1 MODEL RESULTS IN THE ABSENCE OF ATTRACTION, $C = 0$

In the absence of attraction, significant tower formation does not occur, even with relaxed locking and unlocking probabilities. This is discussed above in Section 2.2.2, but we will discuss it further here. Figure S1 shows the effects of unlocking or neighbor-influenced lock probability, with the pure DLA case shown in the boxed-in frame.

The horizontal axis of Figure S1 shows the effects of unlocking: small values of unlocking allow initially fractal structures to break up into smaller aggregations, but too much unlocking leads to no aggregations at all. The effect of decreasing locking factor k_{nl} alone is shown in the left column. This rule modification alone does allow rounder aggregations to form, but the aggregations remain shallow. Without unlocking, it is unlikely that new agents make their way toward the center of the aggregation.

The effects of combining the two rule modifications are discussed above and shown most clearly in the $c = 0$ panels of Fig. 2. No combination of locking and unlocking parameters leads to consistent tower formation. The maximum aggregation size is only 150 individuals and no tower height exceeds 2 agents high. Without attraction, small aggregations are able to form, but none of them come close to approximating the tower formation observed in Figure 1A or those described in Phonekeo et al. (2017).

2 SUPPLEMENTARY MODEL DEFINITIONS

In this section, we provide equations described in the text.

In Section 2, we describe the periodic boundary condition. An agent's position at time step $t + 1$, under the periodic boundary condition, is given by,

$$\mathbf{x}_{t+1} = \mathbf{x}_t + \mathbf{v} \mod L, \tag{S1}$$

where $\mathbf{x}_t$ and $\mathbf{v}_t$ are its position and velocity at time step t. Periodic boundaries are also taken into account when calculating distances between agents, with the distance between positions $\mathbf{x}_1$ and $\mathbf{x}_2$ defined by,

$$d(\mathbf{x}_1, \mathbf{x}_2) = \begin{cases} \|\mathbf{x}_1 - \mathbf{x}_2\| & \text{for } \|\mathbf{x}_1 - \mathbf{x}_2\| \leq \frac{L}{2}, \\ L - \|\mathbf{x}_1 - \mathbf{x}_2\| & \text{for } \|\mathbf{x}_1 - \mathbf{x}_2\| > \frac{L}{2}. \end{cases} \tag{S2}$$

Any two points with a distance further than $\frac{L}{2}$ are closer to the border, and their distance is therefore $L - \|\mathbf{x}_1 - \mathbf{x}_2\|$.

The normalized velocity $\hat{\mathbf{v}}_i$, which defines the agent's motion in Section 2.2.3, is given by,

$$\hat{\mathbf{v}}_i = \underset{\mathbf{u} \in S}{\operatorname{argmax}}\, \mathbf{u} \cdot \mathbf{v}_i, \tag{S3}$$

where S is the set of velocities that reach adjacent pixels in one time step,

$$S = \left\{ \begin{bmatrix} -1 \\ -1 \end{bmatrix}, \begin{bmatrix} -1 \\ 0 \end{bmatrix}, \begin{bmatrix} -1 \\ 1 \end{bmatrix}, \begin{bmatrix} 0 \\ -1 \end{bmatrix}, \begin{bmatrix} 0 \\ 1 \end{bmatrix}, \begin{bmatrix} 1 \\ -1 \end{bmatrix}, \begin{bmatrix} 1 \\ 0 \end{bmatrix}, \begin{bmatrix} 1 \\ 1 \end{bmatrix} \right\}. \tag{S4}$$

The normalized velocity $\hat{\mathbf{v}}_i$ is the adjacent pixel which has the direction closest to the velocity $\mathbf{v}_i$ defined by (2).

3 ALGORITHM SCHEMATIC

Algorithm 1 shows pseudocode for the computational model described in the present work. `rand_order` is a function that randomizes the order of a vector, such as the `randperm` function in MATLAB. $z(target)$ and $z(current)$ refer to the height of the target voxel and the height of the current voxel, respectively. Agents are not allowed to climb more than the height of one agent. They are, however, able to fall by any height (but not below the floor). "Neighbor voxels" refer to the eight voxels that are horizontally adjacent to the target pixel.

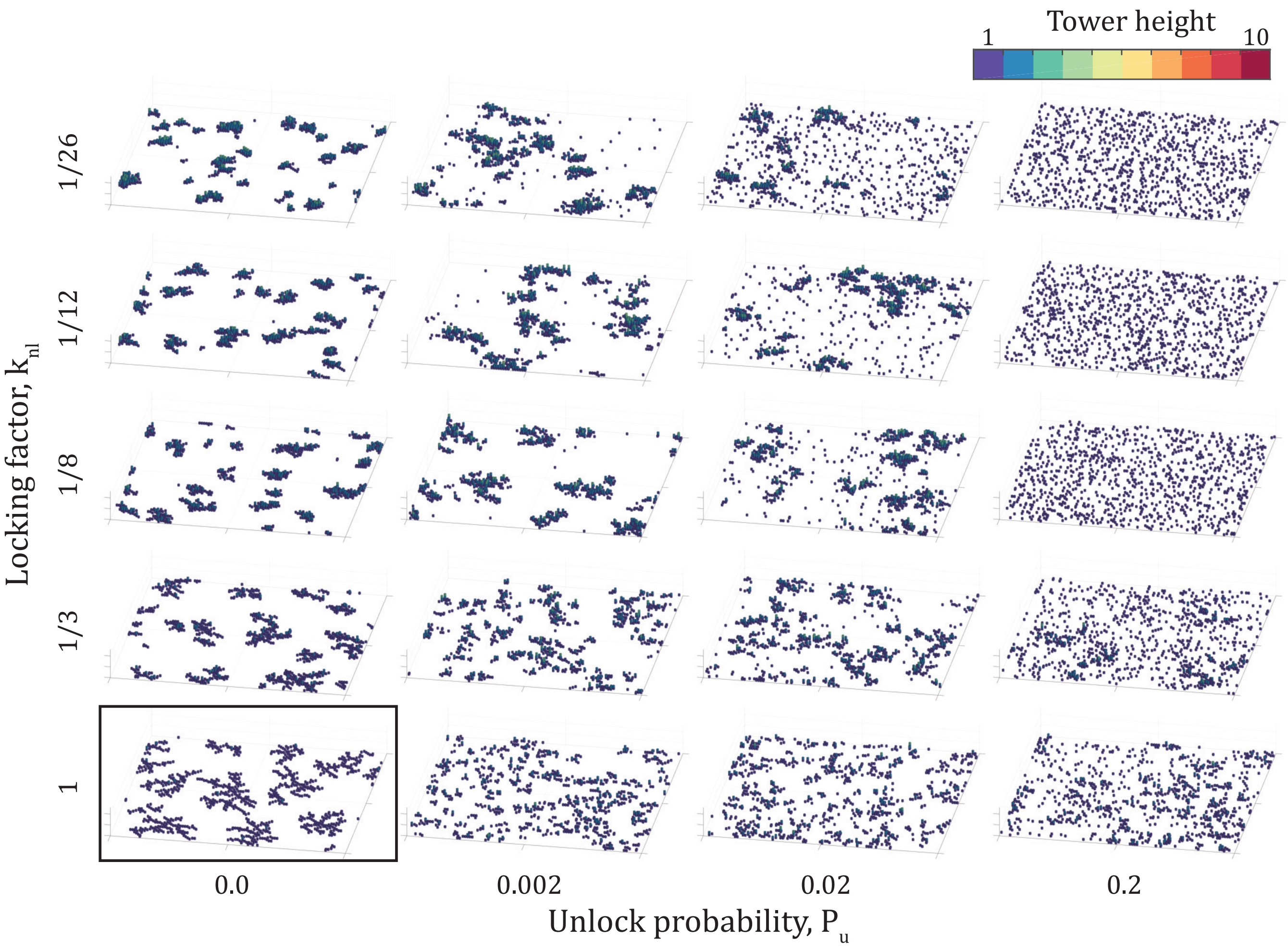

Figure S1: Final configuration of simulations, after 500,000 time steps, incorporating only the effect of unlock probability P_u and neighbor-influenced lock factor k_{nl}. Each panel shows the entire 100×100 arena. The panel surrounded by the box represents the case of no rule modifications, leading to diffusion-limited aggregation.

Algorithm 1: Computational model algorithm

```
Generate random positions;
Calculate inter-agent distances;
for Number of frames do
    for all agents do
        Calculate velocity from neighbor positions;
    end
    for rand_order(all agents) do
        if Not target voxel occupied then
            if z(target) − z(current) ≤ 1 then
                Move to target voxel;
            else
                Stay in current voxel;
            end
        else
            for rand_order(neighbor voxels) do
                if not voxel occupied then
                    if z(target) − z(current) ≤ 1 then
                        Move to neighbor voxel;
                    else
                        Stay in current voxel;
                    end
                end
            end
        end
    end
    Calculate inter-agent distances;
    for all agents do
        if free then
            Lock with probability P_l;
        else if locked then
            if not covered then
                Unlock with probability P_u;
            end
        end
    end
end
```
